# The mRNA expression of SETD2 in human breast cancer: correlation with clinico-pathological parameters

**DOI:** 10.1186/1471-2407-9-290

**Published:** 2009-08-21

**Authors:** W Al Sarakbi, W Sasi, WG Jiang, T Roberts, RF Newbold, K Mokbel

**Affiliations:** 1 St George's University of London, Blackshaw Road, Tooting, London, SW17 OQT, UK; 2 University Department of Surgery, Wales College of Medicine, Cardiff University, CF14 4XN, UK; 3 Institute of Cancer Genetics and Pharmacogenomics, Brunel University, Uxbridge, Middlesex, UB8 3PH, UK

## Abstract

**Background:**

SET domain containing protein 2 (SETD2) is a histone methyltransferase that is involved in transcriptional elongation.

There is evidence that SETD2 interacts with p53 and selectively regulates its downstream genes. Therefore, it could be implicated in the process of carcinogenesis. Furthermore, this gene is located on the short arm of chromosome 3p and we previously demonstrated that the 3p21.31 region of chromosome 3 was associated with permanent growth arrest of breast cancer cells. This region includes closely related genes namely: MYL3, CCDC12, KIF9, KLHL18 and SETD2. Based on the biological function of these genes, SETD2 is the most likely gene to play a tumour suppressor role and explain our previous findings.

Our objective was to determine, using quantitative PCR, whether the mRNA expression levels of SETD2 were consistent with a tumour suppressive function in breast cancer. This is the first study in the literature to examine the direct relationship between SETD2 and breast cancer.

**Methods:**

A total of 153 samples were analysed.

The levels of transcription of SETD2 were determined using quantitative PCR and normalized against (CK19).

Transcript levels within breast cancer specimens were compared to normal background tissues and analyzed against conventional pathological parameters and clinical outcome over a 10 year follow-up period.

**Results:**

The levels of SETD2 mRNA were significantly lower in malignant samples (p = 0.0345) and decreased with increasing tumour stage.

SETD2 expression levels were significantly lower in samples from patients who developed metastasis, local recurrence, or died of breast cancer when compared to those who were disease free for > 10 years (p = 0.041).

**Conclusion:**

This study demonstrates a compelling trend for SETD2 transcription levels to be lower in cancerous tissues and in patients who developed progressive disease. These findings are consistent with a possible tumour suppressor function of this gene in breast cancer.

## Background

Histone-lysine N-methyltransferase (SETD2) is a protein that methylates 'Lys-36' of histone H3. H3 'Lys-36' methylation represents a specific tag for epigenetic transcriptional activation. [1]

We previously demonstrated that the 3p21.31 region of chromosome 3 was associated with permanent growth arrest of breast cancer cells. This region includes closely related genes namely: MYL3, CCDC12, KIF9, KLHL18 and SETD2. Based on the biological function of these genes, SETD2 is the most likely gene to play a tumour suppressor role and explain our previous findings [2].

SETD2 plays a role in chromatin structure modulation during transcriptional elongation via its interaction with hyperphosphorylated POLR2A. It also may act as a transcription activator that binds to promoters. [3]

SETD2 was first implicated in Huntington's disease (HD). Huntington's disease is a neurodegenerative disorder characterized by loss of striatal neurons, is caused by an expansion of a polyglutamine tract in the HD protein Huntingtin. [4] This gene encodes a protein belonging to a class of huntingtin interacting proteins characterized by WW motifs. [5,6] This protein is a histone methyltransferase that is specific for lysine-36 of histone H3, and methylation of this residue is associated with active chromatin. This protein also contains a novel transcriptional activation domain and has been found associated with hyperphosphorylated RNA polymerase II.

It has been established that Histone modifications, which include acetylation, phosphorylation, methylation and ubiquitination, play key roles in gene regulation. [7] These modifications create both agonistic and antagonistic signals that correlate with the transcriptional activity of a gene, through engaging some protein complexes or through changing the structure of chromatin to allow access for RNA polymerase to initiate transcription. [1] These Histone modifications could act as marking system that is responsible for establishing and maintaining programs of gene expression during cellular differentiation.

Previous research has shown that SETD2 could interact with p53 and selectively regulate the transcription factor activity of p53. The interaction was dependent of C-terminal region of SETD2, which contains the SET and WW domains and the N-terminal trans-activation domain of p53 [8]. This evidence demonstrated that SETD2 down-regulated hdm29 (transcription subset gene) expression by targeting its P2 promoter and then enhanced p53 protein stability. These findings suggest that SETD2 could selectively regulate the transcription of subset genes via cooperation with the transcription factor p53. [8] So, hypothetically, increased SETD2 activity could enhance the action of tumour suppressor gene P53 and therefore have tumour suppressive function in human breast cancer.

Furthermore, SETD2 is located on the short arm of chromosome 3 and this region has been associated with permanent growth arrest of the tumour cells. [2]

The aim of our study was to determine, using quantitative PCR, whether the mRNA expression levels of SETD2 were consistent with a tumour suppressive function in human breast cancer. This is the first study in the literature to examine the direct relationship between SETD2 and breast cancer.

## Methods

### Patients & Samples

Institutional guidelines, including ethical approval and informed consent, were followed. Breast cancer tissues (n = 127) and normal background tissues (n = 33) were collected immediately after excision during surgery, and stored at -80°C until use. A consultant pathologist examined hematoxylin and eosin stained frozen sections to verify the presence of tumor cells in the collected samples. Normal tissue was derived from the background breast parenchyma of breast cancer patients within the study group. All tissues were randomly numbered and the details were only made known after all analyses were completed. All patients were treated according to local algorithms of management following a multidisciplinary discussion. Patients treated with breast-conserving surgery received adjuvant radiotherapy. Those with hormone-sensitive malignancy received tamoxifen. Fit patients with node-positive breast cancer or hormone-insensitive large and/or high grade cancer were offered adjuvant chemotherapy. Medical notes and histology reports were used to extract clinico-pathological data (Table 1). A customized database was established to record data.

**Table 1:** Clinical data showing number of patients in each category

Parameter	Category	Number
Node Status	Node positive	65
	Node negative	55
Tumor Grade	1	23
	2	41
	3	56
Tumor Type	Ductal	94
	Lobular	14
	Medullary	2
	Tubular	2
	Mucinous	4
	Others	4
TNM staging	1	69
	2	40
	3	7
	4	4
Clinical Outcome	Disease free	81
	Alive With metastasis	7
	With local recurrence	5
	Died of breast cancer	20
	Died of unrelated disease	7
ER receptor status	ERą negative	26
	ERą positive	62
	ERβ negative	17
	ERβ positive	71

### Materials

RNA extraction kits and reverse transcription kits were obtained from Sigma-Aldrich Ltd (Poole, Dorset, England, UK). PCR primers were designed using Beacon Designer (Palo Alto, CA) and synthesized by Sigma-Aldrich. Custom made hot-start Master-mix for quantitative PCR was obtained from Abgene (Surrey, England, UK). [9,10]

### Tissue Processing, RNA Extraction and cDNA Synthesis

Frozen sections of tissue were cut at a thickness of 5–10 mm and kept for routine histological analysis. Additional 15–20 sections were mixed and homogenized using a hand-held homogenizer in ice-cold RNA extraction solution. The concentration of RNA was determined using UV spectrophotometry. Reverse transcription was carried out using a reverse transcription kit with an anchored olig (dT) primer supplied by Abgene, using 1 mg of total RNA in a 96-well plate. The quality of cDNA was verified using b-actin primers (Appendix 1).

### Quantitative Analysis

The level of SETD2 transcripts from the above prepared DNA were determined using real-time quantitative PCR based on the Amplifluor technology, modified from a method reported previously. [10] PCR primers were designed using Beacon Designer software, but to the reverse primer an additional sequence, known as the Z sequence (5'-ACTGAACCTGACCGTACA-3') which is complementary to the universal Z probe (Intergen, Inc., Oxford, UK) was added. The product expands one intron. The primers used for each SETD2 are detailed in Appendix 1. The reaction was carried out using the following: Hotstart Q-master mix (Abgene), 10 pmol of specific forward primer, 1 pmol reverse primer which has the Z sequence, 10 pmol of FAM-tagged probe (Intergen, Inc.), and cDNA from 50 ng of RNA. The reaction was carried out using the IcyclerIQ (Bio-Rad Ltd, Hemel Heamstead, England, UK), which is equipped with an optic unit that allows real-time detection of 96 reactions, under the following conditions: 94°C for 12 min and 50 cycles of 94°C for 15 sec, 55°C for 40 sec, and 72°C for 20 sec. The levels of the transcript were generated from a standard that was simultaneously amplified with the samples. Levels of SETD2 expression were then normalized against CK19 expression, already measured in these specimens, to correct for varying amounts of epithelial tissue between samples. CK19 transcripts were quantified as previously reported, [11] using primers detailed in Appendix 1. With every PCR run, we included a negative control without a template and a known cDNA reference sample as a positive control.

### Statistical Analysis

The Mann-Whitney U test was used for statistical analysis. SETD2 transcript levels within breast cancer specimens were compared to normal background tissues and analyzed against conventional pathological parameters and clinical outcome over a 10 year follow-up period. In each case the true copy number was used for statistical analysis and hence samples were not classified as positive or negative. Statistical analysis was carried out using Minitab (14.1) using a custom written macro (Stat 2005.mtw).

## Results

The original number studied was 160 samples, but we had to exclude some 7 samples as we had un-interpretable results on analysis possibly due to sample contamination and therefore these samples had to be excluded. SETD2 was found to be expressed in both benign and breast cancer specimens. Quantification of mRNA expression after CK19 normalisation (Table 2) showed that levels of SETD2 were significantly lower in malignant tissue samples compared to normal breast tissue (p = 0.0345). When looking at the median values in table 2 we can also see that levels are more than 23 time lower in malignant samples. This was also apparent when comparing normal tissue to individual subgroups of malignant samples.

**Table 2:** Levels of SETD2 mRNA expression (After CK19 normalisation) in each group of patients

Parameter	subgroups	No.	Median	Mean ± SD
All samples	SETD2 all tissue	153	27	85547 ± 498012
	Normal tissue	33	406	414434 ± 1106629
	Tumor tissue	120	17	20950 ± 37626
NPI	NPI1	68	11	8755 ± 26203
	NPI2	37	24	16517 ± 55868
	NPI3	15	3	318 ± 645
Grade	Grade1	23	3	2592 ± 9686
	Grade2	41	19	11942 ± 35360
	Grade3	56	26	11457 ± 44706
TNM staging	TNM1	69	4	8551 ± 29766
	TNM2	40	42	8578 ± 24043
	TNM3	7	0	174 ± 414
	TNM4	4	221	679 ± 1081
Clinical outcomes	Disease free	81	13	12239 ± 43466
	Alive with Metastasis	7	2	1983 ± 5142
	Local recurrence	5	930	3513 ± 5683
	Died of Br Ca	20	16	698 ± 1741
ER receptor status	ERą negative	7	24	4742 ± 18706
	ERą positive	26	41	14278 ± 46482
	ERβ negative	62	6	1698 ± 4441
	ERβ positive	17	27	13353 ± 43908

Firstly, we examined the correlation between expression levels and tumour stage. We found a marked decrease in SETD2 levels correlating with higher stages of tumour. This was statistically significant when comparing TNM1 to TNM3 (p = 0.038), TNM1 to TNM4 (p = 0.05), and TNM2 toTNM3 (p = 0.05). We then examined the correlation with tumour grade. There was a noticeable trend with lower levels correlating with increasing tumour grade; however, this did not reach statistical significance (p = 0.16 and 0.18 when comparing grade 1 to grade 2 and 3 respectively).

We then examined the correlation with the Nottingham Prognostic Index (NPI) and also noted a distinct decrease in levels with NPI scores. This, however, was only statistically significant when comparing NPI1 to NPI 3 (p = 0.026).

**Figure 1:**
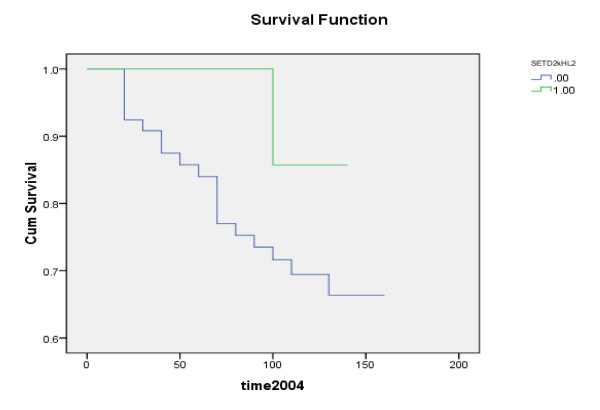
**Kaplan-Meier disease free survival analysis depending on the expression of SETD2**: .00 = low levels. 1.00 = high levels. P = 0.120

**Figure 2:**
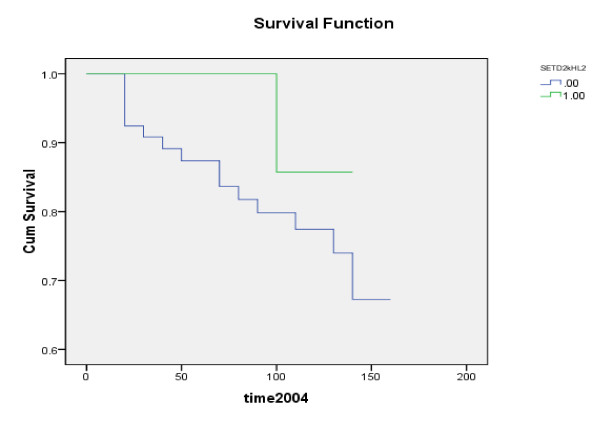
**Kaplan-Meier overall survival analysis depending on the expression of SETD2**: .00 = low levels. 1.00 = high levels. P = 0.232

Furthermore, SETD2 expression levels positively correlated with progression of disease as levels were significantly lower in samples from patients who developed metastasis, local recurrence, or died of breast cancer when compared to those who were disease free for > 10 years (p = 0.041) (median follow up 120 months). Table 3 illustrates the association between levels of expression and disease progression (CK19 normalized). Figure 1 and 2 show Kaplan-Meier survival curves for overall and disease free survival.

**Table 3:** p values for SETD2 mRNA expression levels in relation to survival and disease progression

Parameter	No.	Disease free>10 years
Alive with Metastasis	6	p = 0.062
Local recurrence	4	p = 0.13
Died of Br Ca	16	p = 0.0026
All progressive disease	26	p = 0.041

Data analysis also showed a correlation with ER status (Estrogen receptors in Ductal carcinoma patients). Levels of mRNA were significantly lower in ERb negative samples when compared to positive (p = 0.025). Although data analysis showed no statistically significant correlation between SETD2 expression levels and ERą (p = 0.16).

## Discussion

This is the first study in the literature to examine the direct relationship between SETD2 mRNA expression and breast cancer. Our findings demonstrate that this gene may have a tumour suppressor function and its mRNA expression could play a role as a prognostic indicator in human breast cancer.

SETD2 (SET domain containing protein 2) is a histone H3K36 trimethyltransferase protein that associates with hyperphosphorylated RNA polymerase II and involves in transcriptional elongation.

It has been established that regulation of chromatin structure modulates DNA replication and transcription. One major mechanism that regulates the structure and function of chromatin is the covalent modification of histones, [12,13] and this is linked to SETD2 function. Several studies suggest that SETD2 and modified histones are linked to transcriptional activation and repression, DNA repair, and cell cycle regulation. [14,15]

Previous research showed that SETD2 could interact with p53 and selectively regulate the transcription factor activity of p53. [8] Abnormalities of the p53 tumour suppressor gene are among the most frequent molecular events in human neoplasia. [16] Tumour suppressor p53 acts as transcriptional activator, controlling the expression of a variety of genes important in cell cycle regulation and apoptosis. [17] It is composed of four identical subunits, binds to a specific site on the DNA, and interacts with transcription interaction factors, leading to the initiation of transcription by RNA polymerase II. Furthermore, SETD2 could be co-immunoprecipitated with p53 under both exogenous and endogenous conditions. Therefore, SETD2 over-expression augmented expression of the majority of p53 target genes and SETD2 suppression led to their down-regulation. [8]

Such studies support and explain our finding of a compelling trend for SETD2 mRNA levels to be lower in cancerous tissues and in patients who developed progressive disease (distal metastasis, local recurrence, or died of breast cancer). These observations are consistent with a possible tumour suppressor function of this gene in breast cancer. Furthermore its location on chromosome 3p is also consistent with such possible role. We also demonstrated a statistically significant correlation with ERb status which is a favourable prognostic indicator in human breast cancer.

The strength of our report lies in the use of robust RT-PCR methodology to analyze SETD2 mRNA expression in a cohort of breast cancer patients with a long-term follow up.

## Conclusion

We are the first group to investigate SETD2 expression in human breast cancer and identify a possible TSG function. However there are several inherent limitations to our study related to the relatively small sample size and lack of data regarding protein expression of the gene studied.

Further research is required to confirm the role of SETD2 gene in the pathogenesis of breast cancer including immunohistochemistry studies, in vitro experiments and the preparation of animal models with suppressed SETD2 gene.

If our observations are confirmed by other studies, SETD2 could prove a valuable prognostic marker and its artificial expression could represent a novel therapeutic strategy.

## Competing interests

The authors declare that they have no competing interests.

## Authors' contributions

WAS performed data analysis and manuscript writing. WS performed Data analysis and manuscript writing. WGJ performed study design, data analysis, and manuscript revision. TR performed study design, data analysis, and manuscript revision. RFN performed study design, data analysis, and manuscript revision. KM performed data collection, study design, data analysis, manuscript revision and writing.

## Appendix 1

Primers for SETD2F1

tccaacagtctatggtgtga

Primers for SETDZr1

actgaacctgaccgtacatgagctgtgaaaatctgttg

Primers for SETD2F2

cagaagcttcaggaagagat

Primers for SETD2Zr2

actgaacctgaccgtacattggaagtactttgctttcc

Primers for SETD2F3

gaaaatgccatgttactttga

Primers for SETD2Zr3

actgaacctgaccgtacacacactgcattcgcttaata

Primers for SETD2F4

cccctgaagaagaagaaaat

Primers for SETDZr4

actgaacctgaccgtacaaagcaacacctttgaacatt, setd2Zr4

## Pre-publication history

The pre-publication history for this paper can be accessed here:

http://www.biomedcentral.com/1471-2407/9/290/prepub
